# Importance of medication adherence in treatment needed diabetic retinopathy

**DOI:** 10.1038/s41598-021-98488-6

**Published:** 2021-09-27

**Authors:** Chia-Chen Kao, Hui-Min Hsieh, Daniel Yu Lee, Kun-Pin Hsieh, Shwu-Jiuan Sheu

**Affiliations:** 1 Department of Ophthalmology, Kaohsiung Medical University Hospital, Kaohsiung, Taiwan; 2 Department of Ophthalmology, Kaohsiung Medical University, No. 100, Tzyou 1st Rd., Sanmin Dist., Kaohsiung, 80756 Taiwan; 3 Department of Public Health, Kaohsiung Medical University, No. 100, Tzyou 1st Rd., Sanmin Dist., Kaohsiung City, 80756 Taiwan; 4 Department of Medical Research, Kaohsiung Medical University Hospital, No. 100, Tzyou 1st Rd., Sanmin Dist., Kaohsiung City, 80756 Taiwan; 5 Department of Community Medicine, Kaohsiung Medical University Hospital, No. 100, Tzyou 1st Rd., Sanmin Dist., Kaohsiung City, 80756 Taiwan; 6 Center for Big Data Research, Kaohsiung Medical University, No. 100, Tzyou 1st Rd., Sanmin Dist., Kaohsiung City, 80756 Taiwan; 7 Research Center for Environmental Medicine, Kaohsiung Medical University, No. 100, Tzyou 1st Rd., Sanmin Dist., Kaohsiung City, 80756 Taiwan; 8 School of Pharmacy, College of Pharmacy, Kaohsiung Medical University, Kaohsiung, Taiwan; 9 Department of Pharmacy, Kaohsiung Medical University Hospital, Kaohsiung, Taiwan

**Keywords:** Medical research, Risk factors, Health care, Disease prevention

## Abstract

We aim to investigate the role of medication adherence history in treatment needed diabetic retinopathy (TNDR). We conducted a retrospective nested case–control study using 3 population-based databases in Taiwan. The major one was the 2-million-sample longitudinal health and welfare population-based database from 1997 to 2017, a nationally representative random sample of National Health Insurance Administration enrolled beneficiaries in 2010 (LHID2010). The national death registry and national cancer registry were also checked to verify the information. The outcome was defined as the TNDR. The Medication possession ratio (MPR) was defined as the ratio of total days of diabetes mellitus (DM) medication supply divided by total observation days. MPR ≥ 80% was proposed as good medication adherence. The association of MPR and the TNDR was analyzed. Other potential confounders and MPR ratio were also evaluated. A total of (n = 44,628) patients were enrolled. Younger aged, male sex and patients with less chronic illness complexity or less diabetes complication severity tend to have poorer medication adherence. Those with severe comorbidity or participating pay-for-performance program (P4P) revealed better adherence. No matter what the characteristics are, patients with good MPR showed a significantly lower likelihood of leading to TNDR after adjustment with other factors. The protection effect was consistent for up to 5 years. Good medication adherence significantly prevents treatment needed diabetic retinopathy. Hence, it is important to promote DM medication adherence to prevent risks of diabetic retinopathy progression, especially those who opt to have low medication adherence.

## Introduction

Diabetic retinopathy (DR) has been one of the most common cause of vision loss worldwide, and over one-third of diabetic patients progress to DR^1,2^. It is expected that the number of diabetes patients will have risen to 552 million by 2030, and the increasing prevalence of diabetes mellitus (DM) indicates that more people will suffer from DR in the future^3^.

In DR, early detection and treatment are important in preventing vision loss and blindness^4^. As for DR in the real world, there were several risk factors, including uncontrolled fasting blood sugar, hypertension, longer duration of diabetes, hyperlipidemia, pregnancy, nephropathy, obesity and genetics^5^. Tight glycemic remains the cornerstone in the primary prevention of DR^6^, and poor patient’s diabetic medication compliance was also a critical factor for DR progression^7^. The medication adherence in the early stage of diabetes is important for maximizing the effectiveness of pharmaceutical therapy^8^. Non-adherence to diabetes medication is associated with poor glycemic control, leading to worsened medical conditions and comorbidities, elevated health care costs, and increased mortality.

Recently, patient adherence has gained more and more attention as an important factor for the visual outcome of diabetic complications, such as retinopathy. When retinopathy progresses, it could not be treated with oral medication per se, and treatment for DR mainly includes retina photocoagulation, intravitreal injection as well as vitrectomy. In addition, lost to follow-up was reported by large-scale studies to contribute to visual loss in diabetic patients. Since vision-threatening diabetic retinopathy involves mostly people who work, which cause even more social impact in the country. It is helpful if we can identify the group of patients who opt to have worse compliance and adherence to treatment from the beginning when they visit ophthalmologist. Individualized treatment plan for these patients should help to improve the outcome.

The primary aim of this study was to examine factors associated with end points of receiving DR needed treatment, indicating the worse progression of DR among those patients who were newly diagnosed with DR. Specifically, we focus on the relationship between diabetic medication adherence and treatment needed diabetic retinopathy (TNDR), and we also evaluate if the relationship holds after correction for the confounders. Factors possibly related to diabetic medication adherence were included, such as age, gender, disease severity, and subspecialty in diabetic treatment as well as joining for special care program were investigated.

## Materials, subjects and methods

### Data sources and study design

We conducted a retrospective nested case–control study to examine medication adherence and risks of receiving DR-related treatment among patients with newly diagnosed DR. This study used 3 population-based databases in Taiwan^9^. One database was the 2-million-sample longitudinal health and welfare population-based database of 2010 (LHID2010), which a nationally representative random sample of National Health Insurance Administration (NHIA)-enrolled beneficiaries in 2010, including all updated claims data of those individual random sample since year 1997 to 2017. The LHID2010 provided information on patient comorbid conditions, health provider characteristics, and billing variables to identify treatment procedures^9^. The second was the national death registry, which provides accurate death dates, and causes-of-death information^9^. The third was a national cancer registry, which contains accurate cancer diagnosis data from 1979 through 2017. These databases were encrypted patient identifiers and all data analysis completed during 2020 in the Kaohsiung Branch of the Health and Welfare Data Science Center, the Ministry of Health and Welfare in Taiwan. The hospital Institutional Review Board and Ethics Committee approved and waived informed consent for this study (KMUHIRB-E(I)-20190315), which adhered to the Declaration of Helsinki.

### Study design

We first identified patients with newly diagnosed DR between 2000 and 2017 using diagnosis codes (International Classification of Diseases, Ninth Revision, Clinical Modification [ICD-9-CM] codes 362.0, 362.01-362.07). The first date of newly diagnosed as DR was defined as the entry date. We then excluded patients who were less than 18 years old at entry date, did not receive any antidiabetic medications, had any cancer or death records and had any TNDR prior to the entry date. The case group was DR patients who received DR-related treatment and the control group was DR patients without any DR-related treatment. DR-related treatments were defined as patients of the following treatment codes posts to the first diagnosis date of DR till the study end date, death date, whichever came first. Given treatment codes would be more specific and reflect the severity of DR and the need of treatment in a large database, we used treatment codes to define DR-related treatment, including 60001C (macular photocoagulation 1#), 60002C (macular photocoagulation 2#), 60003C (pan-retinal photocoagulation 1#), 60004C (pan-retinal photocoagulation 2#), 60005C (focal photocoagulation 1#), 60006C (focal photocoagulation 2#), 86206B (simple vitrectomy), 86207B (complicated vitrectomy), 86407B (simple endo-laser 1#), 86408B (complicated endo-laser) or 86201C (intravitreal injection). The index date for the case group was defined as the date of first receiving DR-related treatment, and the index date was assigned to the same pairs of control DR patients without DR-related treatment based on age and gender. Because the baseline characteristics were significantly different between groups, which led to selection bias, we used a 1:1 propensity score matching approach to match cases with comparable controls. The propensity score was generated in a logistic regression with the covariates, including age, gender, chronic illness with complexity index (CIC), and diabetes complication severity index (DCSI). The CIC and DCSI are frequently used in studies^10^. The DCSI includes 7 categories of complications by ICD-9-CM code: cardiovascular complications, nephropathy, retinopathy, peripheral vascular disease, stroke, neuropathy, and metabolic disorders. The CIC index includes non-diabetes physical illness complexity (cancers and gastrointestinal, musculoskeletal, and pulmonary diseases), diabetes-related complexity, and mental illness/substance abuse complexity.

The key exposure variables are the baseline adherence of antidiabetics medications on the basis of the Anatomical Therapeutic Chemical Classification System code (ATC codes A10) from 1 to 5 years prior to the index date based on the MPR. The MPR was defined as the ratio of total days of DM medication supply divided by total observation days. Medication possession ratio (MPR) ≥ 80% was proposed as good medication adherence as MPR ≥ 80% has been proposed as good medication adherence for chronic diseases such as diabetes mellitus and hypertension^11^. The association of MPR and the need for DR treatment was analyzed.

Several potential confounders that may affect outcomes, such as patient demographic covariates, and comorbidities, such as chronic illness with complexity index [CIC], and diabetes complication severity index [DCSI] were investigated. The impact of primary DM treatment provider’s specialties (family medicine and internal medicine, metabolism and endocrinology, cardiology, or others), and the participation of the nationwide diabetes pay-for performance (P4P) program of the patients or the primary health providers were also analyzed.

### Statistical analysis

Pearson’s chi-square test was used to evaluate categorical variables between the case and control groups. The association between medication adherence and the risk of receiving ocular treatment was analyzed by using conditional logistic regression. Potential confounding variables were controlled. Odds ratios (ORs), adjusted ORs (AORs) and 95% confidence intervals (CIs) showed the risk of receiving ocular treatment. “The data analysis for this paper was generated using SAS® software, Version 9.4 of the SAS System for Windows. Copyright ©2020. SAS Institute Inc. SAS and all other SAS Institute Inc. product or service names are registered trademarks or trademarks of SAS Institute Inc., Cary, NC, USA. A *P* value < 0.05 was considered statistically significant.

### Ethical aspects

The Kaohsiung Medical University Hospital Institutional Review Board and Ethics Committee approved and waived informed consent for this study (KMUHIRB-E(I)-20190315), which adhered to the tenets of the Declaration of Helsinki.

Consent to Participate: Since this research was retrospective in design using already existing information, patients’ informed consent was waived.

## Results

### General characteristics of the participants

**Figure 1:**
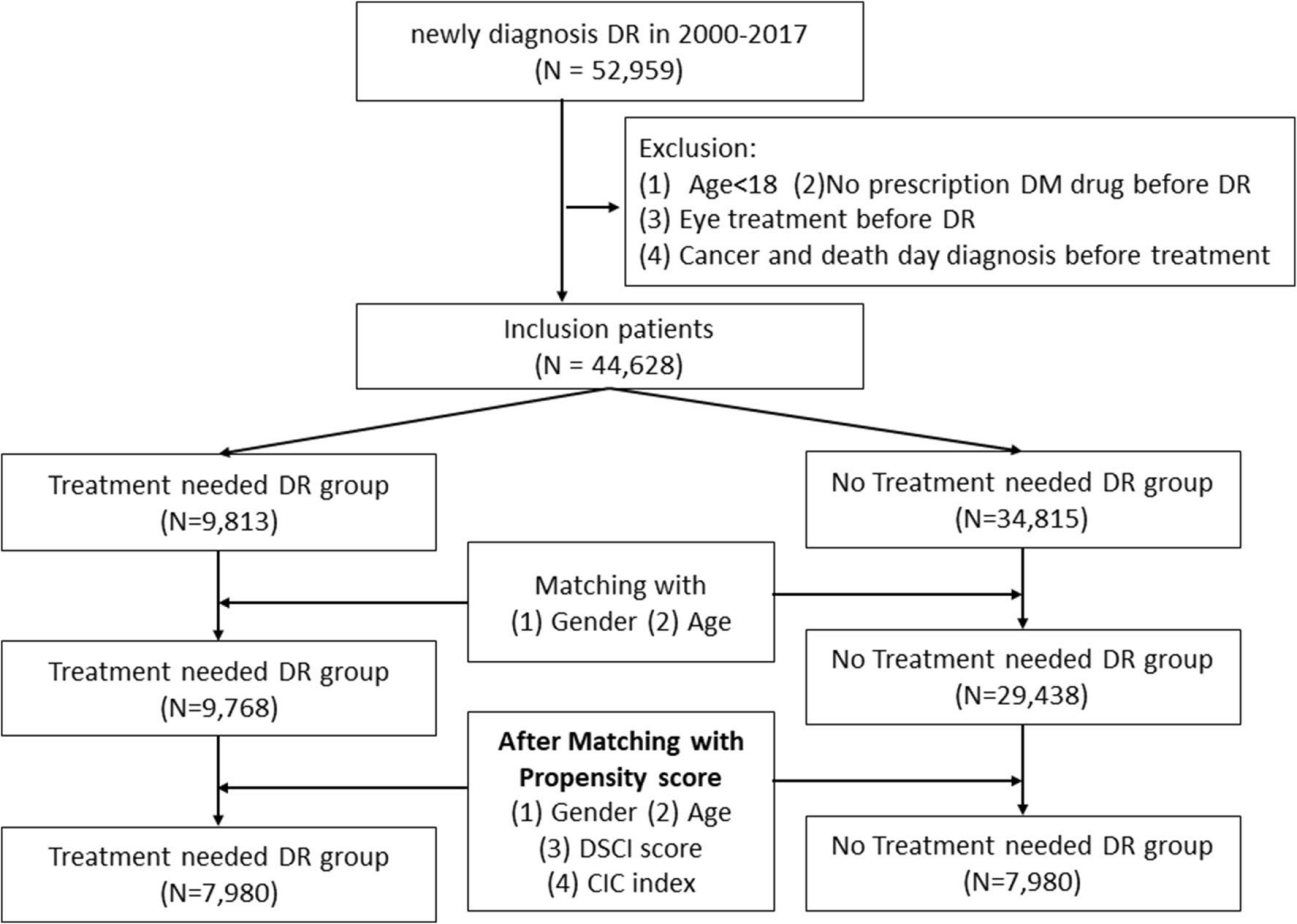
The algorithm of study design. A total of 44,628 patients newly diagnosed with DR between 2000 and 2017 after filtered by the previously mentioned exclusion criteria were included. After matching with propensity score, 15,960 patients were eligible for the final analysis, and there were each 7980 patients in DR treatment group and without DR treatment group, respectively.

A total of 44,628 patients newly diagnosed with DR between 2000 and 2017 after filtered by the previously mentioned exclusion criteria were included. Of these patients, under initial matching with gender and age, 9768 patients received DR treatment and 29,438 patients did not receive treatment for DR. After matching with propensity score, 15,960 patients were eligible for the final analysis, and there were each 7980 patients in DR treatment group and without DR treatment group, respectively (Fig. 1).

After matching with propensity score, between the DR treatment and non-treatment group, there was no significant difference in patient characteristics including age, sex, CIC, DCSI, and leading to availability for comparison between the two groups (Table1).

**Table 1:** Baseline characteristics before and after propensity score matching among the patients with newly diagnosed diabetic retinopathy in 2000–2017.

	Before PS match				After PS match			
	Total	Treatment N(%)	Non-Treatment N(%)	*P*-value	Total	Treatment N(%)	Non-Treatment N(%)	*P*-value
Total	39,206	9768 (24.9%)	29,438 (75.1%)		15,960	7980 (50.0%)	7980 (50.0%)	
**Gender**								
Female	20,665	4894 (50.1%)	15,771 (53.6%)	< 0.0001	8230	4115 (51.6%)	4115 (51.6%)	1
Male	18,541	4874 (49.9%)	13,667 (46.4%)		7730	3865 (48.4%)	3865 (48.4%)	
**Age**								
< 54	11,201	3030 (31.0%)	8171 (27.8%)	< 0.0001	4132	2066 (25.9%)	2066 (25.9%)	1
55–64	12,974	3626 (37.1%)	9348 (31.8%)		5704	2852 (35.7%)	2852 (35.7%)	
65–74	10,643	2388 (24.4%)	8255 (28.0%)		4676	2338 (29.3%)	2338 (29.3%)	
75 ↑	4388	724 (7.4%)	3664 (12.4%)		1448	724 (9.1%)	724 (9.1%)	
**DCSI score**								
0	15,788	749 (7.7%)	15,039 (51.1%)	< 0.0001	1498	749 (9.4%)	749 (9.4%)	1
1–2	15,113	4386 (44.9%)	10,727 (36.4%)		8724	4362 (54.7%)	4362 (54.7%)	
3+	8305	4633 (47.4%)	3672 (12.5%)		5738	2869 (36.0%)	2869 (36.0%)	
**CIC Index**								
0	7929	520 (5.3%)	7409 (25.2%)	< 0.0001	1019	504 (6.3%)	515 (6.5%)	0.0876
1	10,320	2104 (21.5%)	8216 (27.9%)		3940	1964 (24.6%)	1976 (24.8%)	
2+	20,957	7144 (73.1%)	13,813 (46.9%)		11,001	5512 (69.1%)	5489 (68.8%)	
**Department of DM treatment**								
A	15,865	3775 (38.6%)	12,090 (41.1%)	< 0.0001	6270	3174 (39.8%)	3096 (39.8%)	< 0.0001
B	13,288	3648 (37.3%)	9640 (32.7%)		5642	2868 (35.9%)	2774 (35.6%)	
C	2725	506 (5.2%)	2219 (7.5%)		1055	422 (5.3%)	633 (8.1%)	
D	7328	1839 (18.8%)	5489 (18.6%)		2798	1515 (19.0%)	1283 (16.5%)	
**Patient joint P4P**								
No	28,661	6614 (67.7%)	22,047 (74.9%)	< 0.0001	10,965	5610 (70.3%)	5355 (67.1%)	< 0.0001
Yes	10,545	3154 (32.3%)	7391 (25.1%)		4995	2370 (29.7%)	2625 (32.9%)	
**Primary hospital joint P4P**								
No	11,804	1304 (13.3%)	10,500 (35.7%)	< 0.0001	6613	3364 (42.2%)	3249 (40.7%)	< 0.0001
Yes	27,402	8464 (86.7%)	18,938 (64.3%)		9347	4616 (57.8%)	4731 (59.3%)	

*PS* propensity score, *CIC* chronic illness with complexity index, *DSCI* diabetes complication severity index, *DM* Diabetes Mellitus, *P4P* pay for performance, *P* value < 0.05 considered as statistically significant.

Department of DM treatment: (A) Division of Family Medicine & Internal Medicine (B) Division of Metabolism & Endocrinology (C) Division of Cardiology (D) Others.

### Association between baseline characteristics and medication adherence among matched groups

There was a statistically significant correlation between age and MPR in both 1 year and 5 years before the index date, and elder patients have a higher population in the group of MPR > 80% rather than in MPR < 80%. Significant correlation also found between gender and medication adherence, and male patients tend to have lower MPR (AOR = 0.86 in 1 year before the index date and AOR = 0.93 in 5 years before index; AOR < 1 represented a lower ratio to achieve MPR > 80%). Patients with higher scores of CIC index and with the highest DSCI score also revealed significantly better medication adherence. A Significantly higher proportion in the group of MPR > 80% was also noted in patients joint P4P program before DR treatment as well as primary hospital provider joint P4P (Table 2). The trends were similar in both 1 year and 5 years before the index date (Table 2).

**Table 2:** Association between baseline characteristics and medication adherence among the participants one year before index date and five years before index date.

	One year before index date							Five years before index date						
	Total	MPR = > 80	MPR < 80	*P*-value	AOR	95%CI	*P*-value	Total	MPR = > 80	MPR < 80	*P*-value	AOR	95%CI	*P*-value
		N (%)	N (%)						N (%)	N (%)				
Total	14,804	10,221 (69.0%)	4583 (31.0%)					15,018	6717 (44.7%)	8301 (55.3%)				
**Gender**														
Female	7633	5480 (53.6%)	2153 (47.0%)	< 0.0001	1			7725	3657 (54.4%)	4068 (49.0%)	< 0.0001	1		
Male	7171	4741 (46.4%)	2430 (53.0%)		0.86	0.80–0.93	< .0001	7293	3060 (45.6%)	4233 (51.0%)		0.93	0.86–0.99	0.0286
**Age**														
< 54	3792	2142 (21.0%)	1650 (36.0%)	< 0.0001	1			3845	1105 (16.5%)	2740 (33.0%)	< 0.0001	1		
55–64	5281	3653 (35.7%)	1628 (35.5%)		1.72	1.57–1.88	< .0001	5348	2345 (34.9%)	3003 (36.2%)		1.93	1.76–2.11	< .0001
65–74	4362	3304 (32.3%)	1058 (23.1%)		2.34	2.12–2.58	< .0001	4438	2344 (34.9%)	2094 (25.2%)		2.73	2.48–3.01	< .0001
75 ↑	1369	1122 (11.0%)	247 (5.4%)		3.48	2.97–4.08	< .0001	1387	923 (13.7%)	464 (5.6%)		5.13	4.47–5.89	< .0001
**DCSI**														
0	1175	721 (7.1%)	454 (9.9%)	< 0.0001	1			1214	428 (6.4%)	786 (9.5%)	< 0.0001	1		
1–2	8088	5451 (53.3%)	2637 (57.5%)		1.14	0.99–1.32	0.0695	8194	3414 (50.8%)	4780 (57.6%)		1.12	0.97–1.29	0.1173
3+	5541	4049 (39.6%)	1492 (32.6%)		1.25	1.07–1.46	0.0053	5610	2875 (42.8%)	2735 (32.9%)		1.31	1.13–1.53	0.0005
**CIC Index**														
0	821	476 (4.7%)	345 (7.5%)	< 0.0001	1			852	269 (4.0%)	583 (7.0%)	< 0.0001	1		
1	3596	2367 (23.2%)	1229 (26.8%)		1.26	1.07–1.50	0.0069	3663	1420 (21.1%)	2243 (27.0%)		1.24	1.04–1.48	0.015
2	10,387	7378 (72.2%)	3009 (65.7%)		1.35	1.14–1.60	0.0005	10,503	5028 (74.9%)	5475 (66.0%)		1.48	1.24–1.75	< .0001
**Department of DM treatment**														
A	5843	4044 (39.6%)	1799 (39.3%)	< 0.0001	1			5930	2667 (39.7%)	3263 (39.3%)	< 0.0001	1		
B	5411	3810 (37.3%)	1601 (34.9%)		1.00	0.92–1.09	0.9315	5464	2534 (37.7%)	2930 (35.3%)		0.98	0.91–1.07	0.6866
C	952	691 (6.8%)	261 (5.7%)		1.15	0.98–1.34	0.0955	972	452 (6.7%)	520 (6.3%)		1.01	0.88–1.17	0.8759
D	2598	1676 (16.4%)	922 (20.1%)		0.84	0.76–0.93	0.0009	2652	1064 (15.8%)	1588 (19.1%)		0.86	0.78–0.95	0.0021
**Patient joint P4P**														
No	9850	6370 (62.3%)	3480 (75.9%)	< 0.0001	1			10,033	3887 (57.9%)	6146 (74.0%)	< 0.0001	1		
Yes	4954	3851 (37.7%)	1103 (24.1%)		1.92	1.75–2.11	< .0001	4985	2830 (42.1%)	2155 (26.0%)		2.15	1.98–2.34	< .0001
**Primary hospital joint P4P**														
No	5920	3869 (37.9%)	2051 (44.8%)	< 0.0001	1			6039	2406 (35.8%)	3633 (43.8%)	< 0.0001	1		
Yes	8884	6352 (62.1%)	2532 (55.2%)		1.01	0.92–1.10	0.9173	8979	4311 (64.2%)	4668 (56.2%)		1.00	0.92–1.08	0.9437

*CIC* chronic illness with complexity index, *DSCI* diabetes complication severity index, *DM* Diabetes Mellitus, *P4P* pay for payment program, *MPR* medication possession ratio, *AOR* adjusted odds ratio, *95% CI* 95% confidence interval, *P* value < 0.05 considered as statistically significant.

Department of DM treatment : (A) Division of Family Medicine & Internal Medicine (B) Division of Metabolism & Endocrinology (C) Division of Cardiology (D) Others.

### Relationship between medication adherence and treatment needed DR among matched groups

The patients with MPR > 80% had significantly lower likelihood of leading to DR treatment. Consistent results were found in cumulative MPR within different time period prior to the index date (for example, OR 0.65 in 1 year before the index date, OR 0.60 in 2 years before the index date, OR 0.62 in 5 years before index date; OR < 1 represented lower risks to treatment needed DR). The findings indicated that better DM medication adherence showed a protective effect on DR progression to further needs of treatment for DR. The above association was consistent throughout 1 year to 5 years before the index date. Additionally, it also presented similar result after adjustment with other factors, including the department of DM treatment, patient joint P4P program or not before DR treatment and primary hospital provider participating P4P (AOR = 0.68 in 1 year before the index date, AOR = 0.65 in 5 years before index date; AOR (adjusted OR) < 1 represented lower risks to treatment needed DR after adjustment with all confounding factors) (Table 3). Consequently, it implied that higher DM medication adherence was preventive for DR progression and the need of treatment.

**Table 3:** Relationship between medication adherence and treatment needed diabetic retinopathy among the participants one to five years before index date.

	Total	Treatment (N,%)	Non-Treatment (N,%)	OR	95%CI	*P*-value	AOR	95%CI	*P*-value
**1 year before index date**									
MPR < 80%	4583	2802 (35.1%)	1781 (22.3%)	1			1		
MPR > = 80%	10,221	5128 (64.3%)	5093 (63.8%)	0.65	0.60–0.70	< .0001	0.68	0.63–0.73	< .0001
**2 years before index date**									
MPR < 80%	5623	3438 (43.1%)	2185 (27.4%)	1			1		
MPR > = 80%	9288	4512 (56.5%)	4776 (59.8%)	0.60	0.56–0.64	< .0001	0.63	0.58–0.68	< .0001
**3 years before index date**									
MPR < 80%	6681	4047 (50.7%)	2634 (33.0%)	1			1		
MPR > = 80%	8286	3922 (49.1%)	4364 (54.7%)	0.57	0.53–0.61	< .0001	0.60	0.55–0.64	< .0001
**4 years before index date**									
MPR < 80%	7473	4458 (55.9%)	3015 (37.8%)	1			1		
MPR > = 80%	7521	3513 (44.0%)	4008 (50.2%)	0.58	0.54–0.62	< .0001	0.60	0.56–0.65	< .0001
**5 years before index date**									
MPR < 80%	8301	4835 (60.6%)	3466 (43.4%)	1			1		
MPR > = 80%	6717	3140 (39.3%)	3577 (44.8%)	0.62	0.58–0.66	< .0001	0.65	0.60–0.69	< .0001

*MPR* medication possession ratio, *OR* odds ratio, *AOR* adjusted odds ratio, *95% CI* 95% confidence interval.

*P* value < 0.05 considered as statistically significant.

### Subgroups analysis between medication adherence and treatment needed DR among matched groups

**Figure 2:**
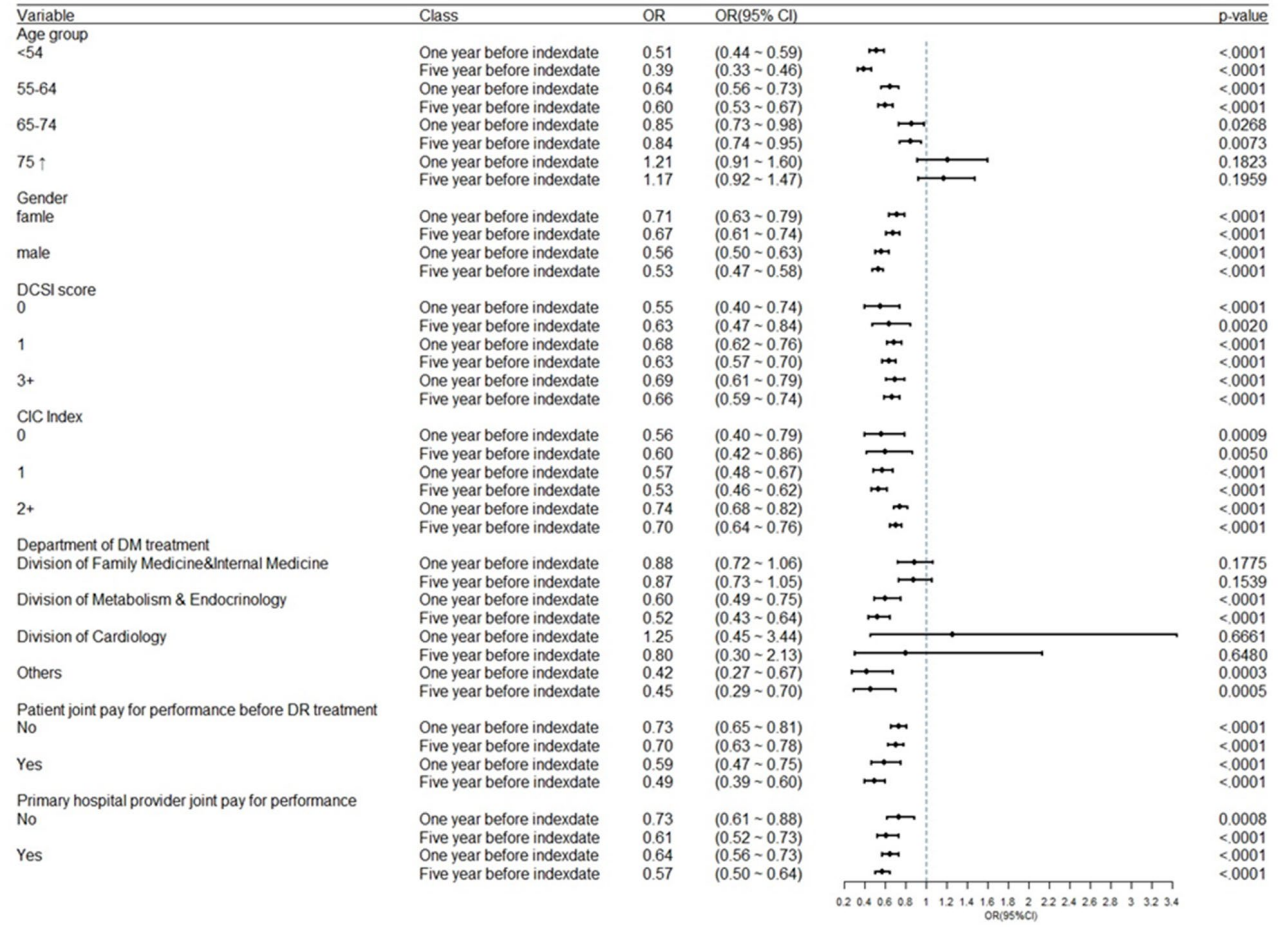
Subgroups analysis between medication adherence and treatment needed DR among matched groups. In each subgroup of the variable factors with gender, DSCI score, and CIC index, patients with MPR > 80% all showed significantly lower likelihood of leading treatment needed DR, and the result was consistent both in 1 year before the index date and in 5 years before the index date. The significantly protective effects existed even if the patients or primary hospitals did not join pay for performance program. (*P* value < 0.05 considered as statistically significant).

In each subgroup of the variable factors with gender, DSCI score, and CIC index, patients with MPR > 80% all showed significantly lower likelihood of leading TNDR, and consistent findings were also found in 1 year before the index date and in 5 years before the index date. A significantly protective effects existed regardless of the participation of P4P program from individual patients or primary hospitals. The significantly protective effects existed even if the patients or primary hospitals did not join P4P program. In general, patients with higher medication adherence showed significantly less need for DR treatment (Fig. 2).

### Limitations

This study had some limitations or exceptions. First, DR was not validated in most of the insurance database studies. Second, there were some unmeasured confounding factors not able to adjust. DM severity was unknown due to a lack of laboratory data, such as HbA1c and, renal function in the NHIRD. These factors were documented as a risk for the development and progression of DR. Some other personal factors (smoking, alcohol consumption, psychologic stress) or medication been actually taken, which might affect the risk of DR, were not known. Although we did match to balance the characteristics, the unmeasured confounding factors might still bias the results.

## Discussion

This study results showed that good medication adherence had a significantly lower risks of leading to DR treatment and the protective effect was consistent for up to 5 years. Elder patients tend to have higher MPR, so did those with higher CIC or highest DSCI. Joining P4P program also helped to achieve better medication adherence. On contrast, male patients tend to have lower MPR. However, the above variables for MPR did not significantly change the protection effect of good MPR.

Adherence to therapies is a primary determinant of treatment success. Failure to adherence is a serious problem that not only affects the patient but also the health care system. Medication non-adherence in patients leads to substantial worsening of disease, death, and increased health care costs. In DR, early detection and treatment are important in preventing vision loss and blindness. DR screening program is taken as an important policy in the management of diabetes in the majority part of the world. Critical factors required for a successful DR screening program is patient adherence to recommendations for follow-up care^12^. Cost and accessibility have been cited as major barriers to eye care adherence by diabetic patients in surveys. But the report showed a low adherence to follow-up appointments in a public clinic with low cost and high accessibility in USA^13^. In Taiwan, National Health Insurance offers low cost and high accessibility of eye care compared to the rest of the world, but the regular follow-up rate is still low^14^. Compared to age-related macular degeneration (AMD), diabetic macular edema (DME) patients have worse compliance and adherence to treatment. In patients with DME, there was a significant correlation between the number of break-offs and change of visual acuity^15^. Lost to follow-up was reported by large-scale studies to contribute to visual loss in diabetic patients. Our study confirmed the protective effect of good medication adherence against TNDR. In our results, TNDR (defined by diagnosis and procedure codes) number should be less than the actual number of DR but more specific for the risk of sight-threatening DR.

The results were compatible with the literature that young age and male sex tend to have lower medication adherence^16^. Those groups might tend to skip medication or return visits due to schedule conflict for work and family or subjective feelings of wellbeing. A study implied that patients typically perceived to be healthy including those who were new to diabetes and on few other medications, may be at risk for non-adherence^12^. Although there were studies showing that patients with comorbidities may have worse medication compliance^12,17^, our subgroup analysis revealed those with high CIC or DCSI had better compliance. This might be due to the wide coverage and extremely low co-payment of our National Health Insurance policy. Patients with comorbidity had greater insight into their disease, hence more frequent hospital visits, and received diabetes treatment as well. As data are shown in Table 1, a certain percentage of patients received diabetes treatment from non-endocrinologists. Those received treatment from others (local health center, but not clinics or hospital) had lower MPR. Again, this variable did not significantly change the protection effect of good MPR. Patient education from healthcare providers is important in improving MPR^18^.

Although our results revealed MPR was affected by several factors, good MPR remained protective from DR treatment after adjusting all the above variables. The positive effect was consistent for up to five years. Based on our data, it is essential to promote DM medication adherence no matter what the patients’ characteristics are. The information about MPR history at the initial visit may help ophthalmologists to arrange an individualized treatment plan for these patients and help to improve the outcome. An intensive treatment plan might be necessary for these patients with low MPR history. More patient education about medication adherence should be emphasized for those who tends to have low MPR, such as male sex, young age, and those not joining the P4P program. To compensate for this difficulty, another option may be e-learning through innovative applications available through smart technologies that can be integrated into a patient’s day to help increase adherence.

This study is the first to evaluate the association between MPR and treatment needed diabetic retinopathy (TNDR). Previous studies showed medication non-adherence leads to substantial worsening of disease, death, and increased health care costs. Large scale studies also showed loss to follow-up contribute to visual loss in diabetic patients. Our results revealed the protective effect of good medication adherence on TDNR, which is more specific for the real threat for vision loss DR. Baseline MPR evaluation might help to improve the cost and effectiveness in the management of diabetic patients.

In conclusion, good medication adherence is essential in the prevention of treatment needed diabetic retinopathy. Several factors, including gender, sex, comorbidity, and joint pay for performance programs play an important role in the prediction of medication adherence. Younger aged, male sex and patients with less chronic illness complexity or less diabetes complication severity tend to have poorer medication adherence. Hence, it is important to promote DM medication adherence to prevent risks of DR progression, especially those opt to have low medication adherence.

## Abbreviations

DR: Diabetic retinopathy
DM: Diabetes mellitus
NHIA: National Health Insurance Administration
TNDR: Treatment needed diabetic retinopathy
DCSI: Diabetes complication severity index
CIC: Complexity index
MPR: Medication possession ratio
P4P: Pay-for-performance program
ORs: Odds ratios (ORs)
AORs: Adjusted ORs
